# Age-associated changes in the heart: implications for COVID-19 therapies

**DOI:** 10.18632/aging.206251

**Published:** 2025-05-13

**Authors:** Colby Wood, Wm. Zachary Salter, Isaiah Garcia, Michelle Nguyen, Andres Rios, Jacqui Oropeza, Destiny Ugwa, Upasana Mukherjee, Ujala Sehar, P. Hemachandra Reddy

**Affiliations:** 1 Department of Internal Medicine, Texas Tech University Health Sciences Center, Lubbock, TX 79430, USA; 2 Nutritional Sciences Department, College Human Sciences, Texas Tech University, Lubbock, TX 79409, USA; 3 Department of Pharmacology and Neuroscience, Texas Tech University Health Sciences Center, Lubbock, TX 79430, USA; 4 Department of Neurology, Texas Tech University Health Sciences Center, Lubbock, TX 79430, USA; 5 Department of Public Health, Graduate School of Biomedical Sciences, Texas Tech University Health Sciences Center, Lubbock, TX 79430, USA; 6 Department of Speech, Language, and Hearing Sciences, Texas Tech University Health Sciences Center, Lubbock, TX 79430, USA

**Keywords:** cardiac aging, mitochondrial dysfunction, oxidative stress, COVID-19 cardiovascular complications, health disparities

## Abstract

Cardiac aging involves progressive structural, functional, cellular, and molecular changes that impair heart function. This review explores key mechanisms, including oxidative stress, mitochondrial dysfunction, impaired autophagy, and chronic low-grade inflammation. Excess reactive oxygen species (ROS) damage heart muscle cells, contributing to fibrosis and cellular aging. Mitochondrial dysfunction reduces energy production and increases oxidative stress, accelerating cardiac decline. Impaired autophagy limits the removal of damaged proteins and organelles, while inflammation activates signaling molecules that drive tissue remodeling. Gender differences reveal estrogen’s protective role in premenopausal women, with men showing greater susceptibility to heart muscle dysfunction and injury. After menopause, women lose this hormonal protection, increasing their risk of cardiovascular conditions. Ethnic disparities, particularly among underserved minority populations, emphasize how social factors such as access to care, environment, and chronic stress contribute to worsening cardiovascular outcomes. The coronavirus disease pandemic has introduced further challenges by increasing the incidence of heart damage through inflammation, blood clots, and long-term heart failure, especially in older adults with existing metabolic conditions like diabetes and high blood pressure. The virus’s interaction with receptors on heart and blood vessel cells, along with a weakened immune response in older adults, intensifies cardiac aging. Emerging therapies include delivery of therapeutic extracellular vesicles, immune cell modulation, and treatments targeting mitochondria. In addition, lifestyle strategies such as regular physical activity, nutritional improvements, and stress reduction remain vital to maintaining cardiac health. Understanding how these biological and social factors intersect is critical to developing targeted strategies that promote healthy aging of the heart.

## INTRODUCTION

Cardiac aging is a complex physiological phenomenon that has garnered significant attention due to its implications for cardiovascular health, overall health, and longevity. The heart, central to the circulatory system, undergoes intricate structural and functional changes over time, influenced by a myriad of genetic, environmental, and physiological factors [1].

The structure and function of the heart are inextricably linked. In youth, the heart is characterized by its efficiency, adaptability, and resilience. However, as age progresses, there is a notable transition toward myocardial hypertrophy, fibrosis, and valvular degeneration, leading to reduced cardiac output and diastolic dysfunction [2]. This evolution of structure and function through a variety of biochemical and cellular processes underscores the heart’s vulnerability to age-related diseases and conditions. Gender plays a pivotal role in cardiac aging. Women, shielded by the benefits of estrogen, often experience a delayed onset of cardiac aging. However, in post-menopause, this protective effect diminishes, emphasizing the hormonal influence on cardiac health [3]. Furthermore, ethnic orientation influences cardiac aging. Certain ethnicities, due to genetic predispositions and cultural lifestyle factors, may be more susceptible to specific cardiac pathologies, necessitating tailored cardiac care approaches [4]. Regarding metabolic dysfunction, diabetes has become increasingly associated with major cardiovascular diseases [5].

**Figure 1:**
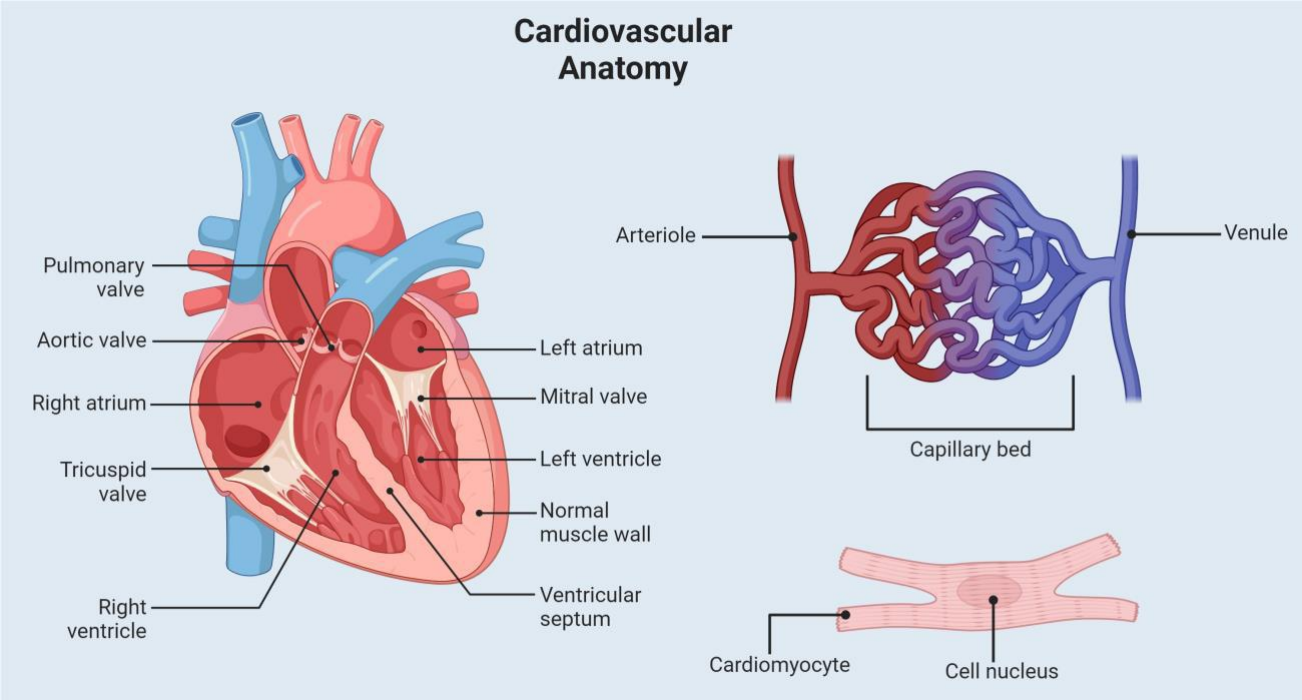
**Cardiovascular anatomy.** This figure shows a visual representation of some of the relevant cardiovascular anatomy. Labeled structures include the heart chambers, valves, capillary system, and a cardiac muscle cell.

The recent COVID-19 pandemic has added another layer of complexity. Preliminary studies suggest that the virus might accelerate cardiac aging due to myocardial inflammation and stress [6]. Acute respiratory infections pose a heightened risk of cardiovascular death, particularly in the weeks following infection, especially among elderly individuals and those with preexisting cardiovascular conditions. The severity of pneumonia in such patients is directly correlated with an elevated risk of mortality [7]. Elderly individuals with preexisting CVD often face heightened complications post-COVID-19 infection, including increased risk of cardiovascular events and mortality, underscoring the need for specialized care and monitoring in this vulnerable population, refer to Figure 1. Moreover, the pandemic has highlighted the importance of considering gender diversity, ethnic disparities, and socio-economic circumstances as crucial determinants of health, especially in the context of cardiovascular diseases [8]. Despite localized reductions in CVD prevalence, CVD remains a leading cause of global morbidity and mortality. The full impact of the COVID-19 pandemic on global health is still unfolding, but its effect on cardiovascular health is undeniable [9]. In the United States, the incidence of CVD, especially stroke and heart failure, is expected to rise dramatically from 2025 to 2060, alongside increasing risk factors like diabetes, obesity, hypertension, and dyslipidemia [10]. This surge will disproportionately affect racial and ethnic minority populations who face greater healthcare access challenges and social determinants of health barriers, highlighting social injustices. In South America, CVD-related deaths accounted for approximately 60% of all-cause mortality between 2000 and 2020 [11]. In Europe, over 113 million people live with CVD, the leading cause of death, costing over €210 billion annually [12]. Sub-Saharan Africa has seen a >50% increase in CVD-related deaths over the past three decades, with hypertension rates the highest globally [13]. In the Middle East, rising non-communicable diseases, including CVD, are linked to lifestyle changes due to economic development. Asia, despite economic growth, continues to face significant healthcare disparities [14]. In Australia, CVD causes 26% of deaths annually, with substantial economic costs [15]. The COVID-19 pandemic has further strained global health systems, disrupting economies and healthcare priorities [9].

The COVID-19 pandemic has significantly contributed to the global burden of CVD. Studies reveal that individuals with pre-existing CVD are at heightened risk for severe outcomes from COVID-19, including hospitalization, intensive care unit admission, and death. CVD was one of the leading causes of death globally, accounting for over 18 million deaths in 2019 [16]. The pandemic exacerbated this burden, with COVID-19 infections precipitated severe cardiovascular events, leading to increased mortality rates among individuals with heart disease [17]. Excess deaths in CVD patients during the pandemic were particularly pronounced due to disruptions in medical care, delayed treatments, and complications arising from COVID-19 infection itself [18]. Additionally, a significant rise in CVD-related deaths across Europe as a direct consequence of the pandemic, underscoring the widespread impact on public health systems was observed [19]. This global surge in cardiovascular mortality emphasizes the need for targeted healthcare strategies to address the compounded risks posed by COVID-19 on CVD patients [20].

COVID-19 has been shown to exacerbate oxidative stress and mitochondrial dysfunction, both of which are critical factors in the progression of cardiac aging. The viral infection directly impairs mitochondrial function, leading to increased production of reactive oxygen species (ROS), which causes oxidative damage to cellular components [21]. Mitochondrial dysfunction in COVID-19 patients may contribute to heart failure by impairing energy production, thereby weakening myocardial performance. Furthermore, the interaction between COVID-19-induced mitochondrial damage and the immune response plays a significant role in accelerating aging processes, particularly in the cardiovascular system [22]. Mitochondrial-based immunity, which typically declines with age, is further compromised in the context of COVID-19, leading to an increased risk of inflammation and subsequent cardiovascular degeneration [23]. Chronic oxidative stress and mitochondrial dysfunction are also central to the development of long-term cardiac complications, suggesting that long COVID-19 may involve persistent mitochondrial damage that impairs heart tissue regeneration [24]. Additionally, individuals with underlying mitochondrial disorders, particularly those in vulnerable populations, are more susceptible to severe cardiovascular consequences of COVID-19, further accelerating cardiac aging. These findings underscore the interconnectedness of mitochondrial health, oxidative stress, and cardiac aging, exacerbated by the effects of COVID-19 [25].

Addressing cardiac aging requires a multifaceted approach. Therapeutic strategies, ranging from pharmacological interventions to cell therapies, offer promise in mitigating age-related cardiac decline [26]. Concurrently, lifestyle changes, encompassing a balanced diet, regular exercise, and stress management, are pivotal in enhancing cardiac health span [27]. Understanding the myriad factors influencing cardiac aging is paramount for developing targeted interventions. The purpose of this article is to examine the impact of COVID-19 on heart health, particularly its effects on the aging heart. Additionally, we aim to investigate the long-term implications of COVID-19 infection on the cardiac health of the aging population. Through a comprehensive exploration of cardiac aging, encompassing structural, functional, and external influences, we seek to promote cardiac health in aging individuals. Furthermore, we delve into the role of inflammation, oxidative stress, and mitochondrial function/dysfunction in the aging heart. Additionally, our article addresses the influence of gender on cardiac aging and proposes distinct therapeutic approaches tailored for male and female-aged individuals, while considering age-related diseases.

## Structure and function

The cardiovascular system, often referred to as the body’s “lifeline,” plays a crucial role in ensuring the optimal functioning of all bodily tissues. Its primary responsibility is the efficient transportation of blood throughout the body [28]. This transportation is vital as it ensures that cells receive the essential nutrients they require for metabolic processes and simultaneously facilitates the removal of cellular waste products [28]. Central to this system is the heart, a robust muscular organ that acts as a pump. Through its rhythmic contractions, the heart generates the necessary pressure to propel blood through an intricate network of blood vessels (Figure 1). The myocardium, the heart’s muscular layer, is specially designed for endurance and is made up of interconnected cardiomyocytes to allow for coordinated contractions [29]. This unique arrangement is coupled with the heart’s intrinsic electrical conduction system to ensure a rhythmic and efficient heartbeat. Blood is supplied to the heart via the coronary arteries and veins which play a pivotal role in ensuring the heart receives the oxygen and nutrients it needs to function optimally [29].

The arteries, which are robust and elastic vessels, are responsible for carrying oxygen-rich blood from the heart to various tissues [28]. Due to their role in withstanding the high pressure generated by the heart’s contractions, they house only a small fraction of the body’s total blood volume [28] (Figure 1). Veins, characterized by their larger diameter and thinner walls, transport oxygen-depleted blood from the tissues back to the heart. Operating under lower pressure, they accommodate the majority of the body’s blood volume [28]. Bridging the arteries and veins are the capillaries, delicate and thin-walled vessels that permeate tissues. It is within these capillaries that the critical exchange of nutrients, waste products, and fluids takes place, ensuring that cells receive what they need and are rid of what they do not [28]. Beyond its primary function of blood transportation, the cardiovascular system is intricately linked to various homeostatic processes that maintain the body’s internal equilibrium. It also exhibits remarkable adaptability, responding to various physiological challenges such as blood loss (hemorrhage), physical activity (exercise), and even shifts in body position, ensuring that the body’s needs are consistently met.

## Evolution of structure and function

The heart, a marvel of biological engineering, has undergone a transformative journey over eons. Its evolution, from rudimentary contractile systems in primitive organisms to the intricate multi-chambered structure in mammals, is a testament to nature’s adaptability. From primordial beginnings to advanced mammalian design, the heart’s evolutionary trajectory traces its lineage from the most basic life forms to the complexity of humans [30]. Early multicellular organisms, around 800 million years ago, featured a primitive coelom surrounded by endoderm, serving passive roles in respiration, nourishment, and reproduction [30]. Over time, the specialization of this “gastroderm” led to the emergence of mesoderm in Bilateria (organisms with bilateral symmetry as developing embryos), giving rise to the initial cardiac myocytes [30]. A “gastrovascular” formation appeared and this structure’s evolution in bilaterian offshoots like Ecdysoa (Drosophila) and Deuterostoma (amphioxus) eventually led to a basic tubular heart devoid of valves, blood vessels, or even blood, but with a singular contracting mesodermal layer [30]. The emergence of Chordata saw this rudimentary heart undergo significant transformations: it looped, established a one-way circulation, developed a closed vascular system, and introduced a conduction mechanism [30]. In mammals, the heart has reached a pinnacle of complexity, with four chambers that ensure efficient separation and circulation of oxygenated and deoxygenated blood.

## Mechanism of cardiac aging

Cardiac aging happens due to several factors that change how the heart works over time. As we age, some heart cells become less effective and can cause inflammation [31]. Increased levels of harmful molecules can damage heart cells, and over time, the heart tissue can become stiffer due to more collagen buildup. Mitochondria, which are the energy producers in cells, may not work as well, leading to cell death [32]. Additionally, low-level inflammation can worsen heart health, and aging can disrupt how calcium moves in and out of heart cells, reducing their ability to contract and relax properly. Together, these changes make older adults more vulnerable to heart diseases [33]. The heart’s aging process is a multifaceted phenomenon driven by a combination of genetic factors, environmental exposures, and intrinsic cellular changes. As the heart ages, it undergoes a series of complex phenotypic changes that can compromise its function and increase the risk of cardiovascular diseases (CVD) [34]. These changes encompass pathological myocardial remodeling, left ventricular systolic and diastolic dysfunction, cardiac hypertrophy, arrhythmia, microcirculatory dysfunction, and heart failure (HF) [35]. Such biological alterations can lead to a decline in cardiac function, making the heart more susceptible to stress. Consequently, the heart’s vulnerability significantly elevates the risk of CVD [36]. This heightened risk manifests as an increased incidence of conditions like coronary heart disease, myocardial infarction (MI), stroke, and atherosclerosis, all of which become more prevalent with advancing age [35]. While aging per se does not directly induce HF, it does decrease the threshold for the disease to manifest. As the average age of populations in most developed nations rises, the significance of aging as a risk factor for all CVD correspondingly escalates. In the United States, for instance, it’s projected that by 2030, the population over 65 years old will reach approximately 70 million, constituting nearly a quarter of the total population [37]. As individuals age, there’s a noticeable decline in physiological reserves referred to as homeostenosis. This depletion makes older adults intolerant to further challenges, leading to frailty once the reserves are exhausted. Yet, without challenges, many age-related changes remain clinically silent. These reserves are partly utilized for maintaining homeostasis, and measuring their usage, termed allostatic load, can predict age-related outcomes. Low-level inflammation associated with age may drive many of these processes. The vast heterogeneity in how aging is experienced, including differences between men and women, contributes to the nonspecific presentations of illness among older adults, reflecting the variability in the loss of reserves [38].

**Figure 2:**
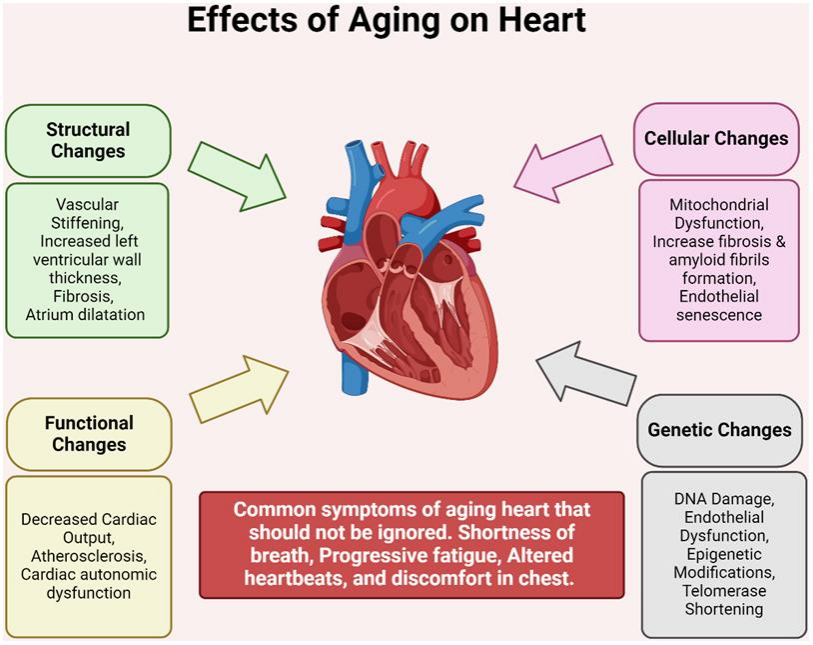
**Effects of aging on heart.** As the heart ages, it experiences a range of structural, functional, cellular, and genetic changes. Structural alterations include vascular stiffness, thickening of the left ventricular wall, fibrosis, and enlargement of the atrium. Functional changes manifest as reduced cardiac output, atherosclerosis, and dysfunction in cardiac autonomic regulation. At the cellular level, there’s mitochondrial dysfunction, heightened fibrosis, amyloid fibril formation, and endothelial senescence. Additionally, genetic alterations such as DNA damage, endothelial dysfunction, and telomerase shortening occur with age.

As the heart ages, it undergoes a multitude of changes across its structure, function, cellular composition, and genetics (Figure 2). Structural shifts involve increased vascular stiffness, thickening of the left ventricular wall, fibrosis, and enlargement of the atrium. Functional adjustments result in diminished cardiac output, atherosclerosis, and disruptions in cardiac autonomic regulation. On a cellular level, there’s observed mitochondrial dysfunction, heightened fibrosis, formation of amyloid fibrils, and endothelial senescence. Moreover, genetic modifications, including DNA damage, endothelial dysfunction, and telomerase shortening, are evident with aging.

## Impaired cellular autophagy

Autophagy describes a cellular “self-consuming” process that plays a pivotal role in maintaining cellular homeostasis [39]. This lysosomal-dependent biological process is responsible for the degradation and recycling of long-lived or misfolded proteins and damaged organelles ensuring the renewal of organelles and supporting cellular metabolism [28]. Autophagy’s role in cellular health is multifaceted. On one hand, it contributes to cell survival by eliminating damaged organelles or cellular components. On the other, excessive autophagy can promote cell death in many physiological and pathological conditions [28]. This duality is particularly evident in the context of cardiac aging. Autophagy is crucial for maintaining cellular and protein homeostasis in cardiomyocytes, and it plays a vital role in reducing cardiac injury and preserving cardiac function during the aging process [40]. However, the aging heart has been found to display decreased autophagic activity with time [40]. In a study done by Taneike et al., a heart-specific knockdown of the Atg5 gene drastically reduced mitochondrial respiration and hastened left ventricular hypertrophy and cardiac aging in mouse models when compared to wild type controls [41]. Structural analysis of these mice displayed chaotic sarcomere structure and mitochondria that had collapsed resulting in decreased aerobic respiration functions [41]. According to the above studies, cardiac aging is marked by several pathological changes, including myocardial hypertrophy, fibrosis, accumulation of misfolded proteins, and mitochondrial dysfunction all of which deficient autophagy plays a major role [40–42]. Despite the mounting evidence underscoring the significance of autophagy in cardiac aging, the therapeutic potential of enhancing autophagy in patients with cardiac aging remains an area of active research.

## Oxidative stress in cardiac aging

Oxidative stress plays a pivotal role in the aging process of the heart. At the molecular level, free radicals, particularly ROS, are atoms or groups known for their strong oxidizing characteristics [36]. Due to their high reactivity and instability, ROS can easily interact with other molecules, leading to cellular damage [43]. Harman’s free radical theory of aging, proposed in 1956, suggests that endogenous free radicals, originating from oxidation-reduction reactions during fundamental metabolic processes, contribute to aging-associated diseases by exerting long-term harmful effects on cells and tissues [36, 43]. Cardiomyocytes, the muscle cells of the heart, have high energy requirements and to meet these demands, the heart has a higher rate of oxygen consumption compared to other tissues, leading to increased ROS production [28, 44]. ROS have been implicated in the development of several cardiovascular diseases, such as hypertension, atherosclerosis, cardiac hypertrophy, and heart failure [45]. Recent studies have highlighted the profound effects of oxidative stress on cardiac aging. Oxidative stress can activate the TGF-β pathway, leading to the accumulation of miR-29, which contributes to cardiac aging. Blocking this pathway can improve cardiac function in aging mice, suggesting its potential as a therapeutic target [46]. Enzymes that are associated with oxidative reactions, such as mitochondrial superoxide dismutase (SOD) and monoamine oxidase (MAO), also aggravates cardiac aging [47]. Notably, MAO, located on the outer mitochondrial membrane, catalyzes the oxidative deamination of substrate monoamine, producing hydrogen peroxide and contributing to intracellular ROS production [36].

While the role of oxidative stress in cardiac aging is evident, the use of antioxidants as a therapeutic intervention has been met with mixed results. Some studies have suggested that low concentrations of ROS might play a protective role by triggering defense mechanisms against cell damage this has been termed mitohormesis and suggests that while high levels of free radicals are associated with cell injury, a moderate increase can stimulate cells to enhance protective mechanisms [48]. Contrary to the traditional view that ROS are harmful, mitohormesis posits that low ROS concentrations are essential for cell function and health, mediating vital signaling pathways that support cell survival and proliferation [48].

## Mitochondrial dysfunction in cardiomyocytes

Mitochondria, often referred to as the “powerhouses” of the cell, play a pivotal role in cellular energy production and homeostasis [28]. These semiautonomous organelles, encapsulated by two layers of membranes, are the primary sites for aerobic respiration and ATP generation in eukaryotic cells [28]. With age, the functionality of these organelles can be compromised, leading to a cascade of detrimental effects on the cell [49]. One of the hallmarks of this dysfunction is the accumulation of mutations in mitochondrial DNA, which encodes essential components of the electron transport chain. As age progresses, point mutations and deletions in mitochondrial DNA increase, especially harming tissues with high energy demands like the heart [49]. When mitochondria or mitochondrial DNA are damaged by internal or external stimuli, there’s an imbalance between oxidative stress and antioxidation, leading to increased ROS production and genomic instability [50].

The intricate relationship between oxidative stress and mitochondrial dysfunction in cardiac aging is evident. To further exacerbate this aging process, it is well known that cardiomyocytes have the most plentiful mitochondria of all bodily cells [51]. The inability to implement suitable modifications in mitochondrial characteristics could potentially disrupt the metabolism of energy substrates or hasten the production of ROS [51]. This is crucial as mitochondria play a pivotal role in energy production, and any imbalance can lead to cellular dysfunction and increased oxidative stress. In the landscape of hypertension and cardiac hypertrophy, the shadows of mitochondrial alterations loom large. These conditions exhibit clear-cut changes in both the quantity and quality of mitochondria, mirroring the compromised state of these organelles and their contribution to the exacerbation of these cardiac conditions [51]. These changes can contribute to the progression of cardiac conditions by impairing the energy supply necessary for heart function and increasing oxidative damage to cardiac cells. The resulting mitochondrial dysfunction is a significant factor in the pathophysiology of hypertension and cardiac hypertrophy, underscoring the importance of maintaining mitochondrial health for optimal cardiac function [51].

The exploration of mitochondrial dynamics in the context of cardiac health and aging is not just a scientific endeavor but a pressing imperative. A deeper, more nuanced understanding of these aspects will not only enrich the academic dialogue around cardiac aging but also pave the way for the development of innovative, targeted therapeutic strategies. These approaches, anchored in the enhancement and preservation of mitochondrial function, could emerge as potent shields against the onslaught of cardiac aging, hypertension, and hypertrophy, bolstering cardiac health and longevity amidst the challenges of aging.

## Gender and cardiac aging: a comprehensive analysis

**Figure 3:**
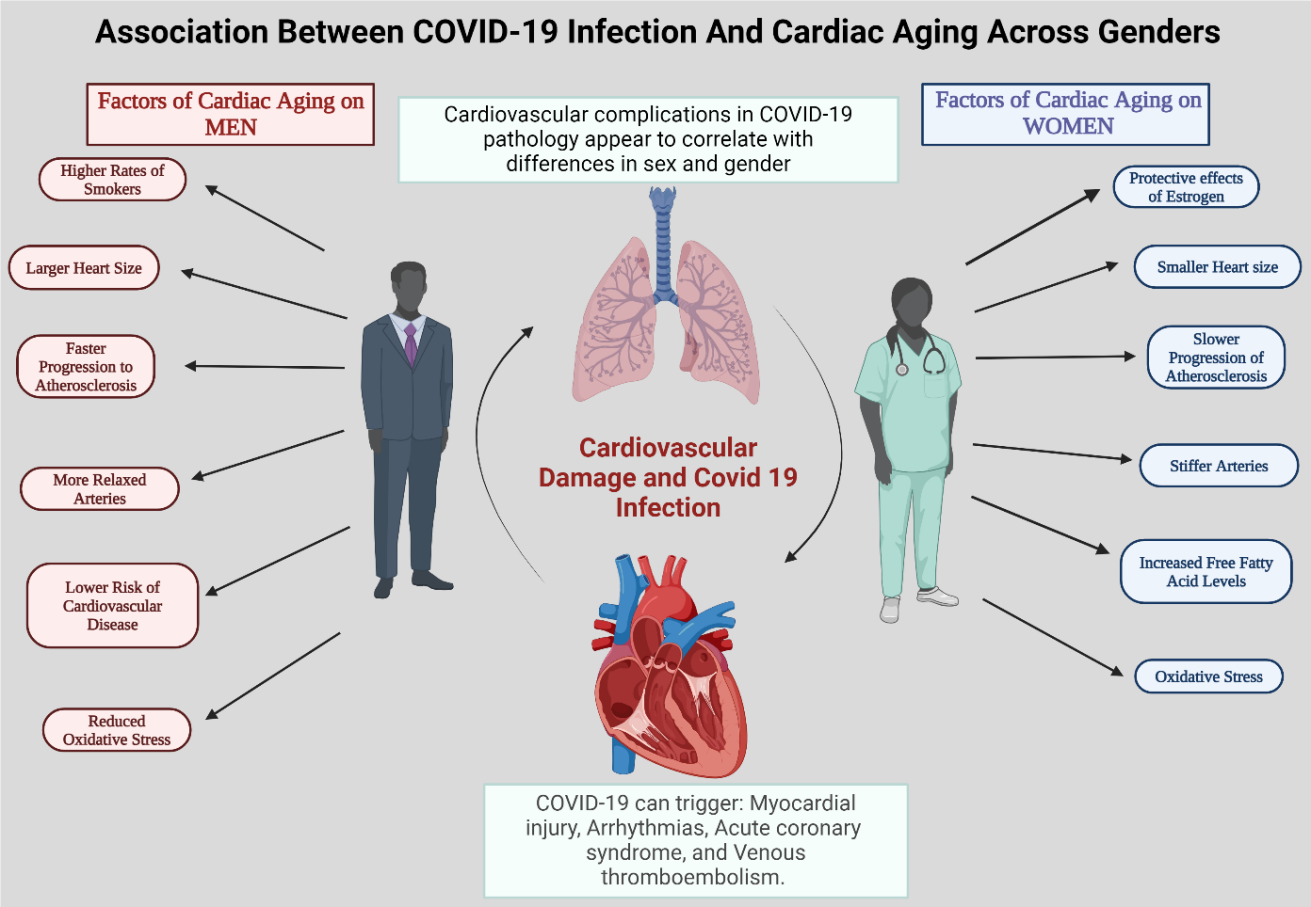
**A summary of different factors that contribute to the differences in cardiac agedness in men and women.** The incidence of cardiovascular complications in COVID-19 pathology appears to be linked to differences in sex and gender. This likely contributes to the greater severity and poorer outcomes of SARS-CoV-2-mediated disease in male patients compared to females.

COVID-19, stemming from the SARS-CoV-2 virus, impairs older individuals, especially men, ethnic minorities, and those with underlying conditions like compromised immunity, cardiovascular disease, and diabetes, disproportionately. The variation in COVID-19 incidence and severity is multifaceted, likely influenced by biological, social, and nutritional factors [52]. Cardiovascular diseases, whether pre-existing or developing during infection, play a pivotal role in determining COVID-19 outcomes, with differing impacts observed between men and women [53]. While COVID-19 is primarily a severe respiratory illness, it often leads to acute myocardial injury, as indicated by elevated levels of high sensitivity cardiac troponin I (cTnI) or cardiac troponin T (cTnT) in up to 28% of confirmed cases [54]. Artico and colleagues study showed that in contrast to contemporary controls, individuals with COVID-19 and heightened cardiac troponin levels exhibit greater ventricular impairment and myocardial scarring in early convalescence. Nevertheless, the incidence of myocarditis was minimal, and scar development varied, encompassing a newly identified pattern of microinfarction [55]. This myocardial injury is linked to significantly worse outcomes, with mortality rates increasing 7- to 11-fold. Patients with pre-existing cardiovascular disease and elevated TnT levels face the highest mortality rates [56]. Pre-existing cardiovascular disease (CVD) appears to correlate with more severe outcomes and a higher risk of death among patients with COVID-19 [57]. Additionally, COVID-19 can trigger myocardial injury, arrhythmias, acute coronary syndrome, and venous thromboembolism [58]. While a plethora of factors such as smoking have been firmly established as risk factors for cardiovascular disease, an escalating acknowledgment of gender’s significant impact on cardiovascular system aging is emerging [59]. These gender-related cardiac differences, discernible early in life, have the potential to exert profound effects in later years. Despite overarching similarities in cardiac aging across genders, several crucial differences prevail (Figure 3).

The potential variations based on sex, gender is a multifaceted social construct encompassing social roles, identities, and relationships, which may impact exposure to and risk of infectious diseases. For instance, epidemiological evidences suggest a potential gender predisposition to COVID-19, with men being more prone to experiencing severe manifestations, and among older individuals, men constitute the majority of fatalities [60], possibly due to men’s higher prevalence of risky behaviors such as smoking and alcohol consumption. Moreover, men are more likely to work in high-risk occupations, such as transportation, which increases their likelihood of exposure to the infection or delays in seeking medical attention when symptoms worsen. These factors could, at least partially, explain the greater severity of infections and subsequent outcomes in men [61].

Age is not only a risk factor for CVD but age-related changes in the immune system, known as immunosenescence, are widely acknowledged as the primary cause of heightened susceptibility to infections, notably respiratory ones like influenza, and diminished response to vaccinations. Chen and colleagues suggested an immunological explanation for the vulnerability of older adults to COVID-19. This explanation revolves around the age-related decline in immune defenses against SARS-CoV-2, termed immunosenescence, and the heightened risk of immune-related pathology [62]. Patients with COVID-19 often experience cardiovascular complications, which can stem from either systemic and cardiac inflammation associated with the virus or a hypercoagulable state induced by the virus itself [63]. Another challenge faced by older patients with CVD is their reluctance to seek medical care due to concerns about contracting COVID-19. It’s important to note that hypertension, diabetes, and obesity, frequently associated with CVD, are already established as risk factors for severe COVID-19, necessitating vigilant management [64].

Aging leads to ventricular hypertrophy, fibrosis, and changes in how the heart functions, but these processes differ between men and women. In older men, the heart often shows eccentric remodeling, systolic dysfunction, and reduced sensitivity to adrenergic signals. In contrast, older women tend to experience diastolic dysfunction and concentric remodeling. While some of these changes may be linked to fluctuations in sex hormones, others are likely influenced by non-hormonal factors or occur at different times in men and women [65]. The impact of biological sex on heart disease has been recognized for some time. Men have a higher incidence of hypertension, which increases in women after menopause. Premenopausal women are generally at lower risk for cardiovascular disease compared to age-matched men, but this advantage diminishes after menopause, indicating that sex hormones boosts blood pressure regulation. Additionally, the heart undergoes remodeling throughout life, with different patterns observed in men and women. Premenopausal women tend to have lower autonomic tone, better baroreceptor response, and improved vascular function compared to men of the same age. However, after menopause, women experience stiffer arteries than men [66].

Hormones, especially estrogen, play an indispensable role in cardiac aging. The cardioprotective attributes of estrogen elucidate the generally lower risk of CVD in premenopausal women compared to their male counterparts [67]. Nevertheless, the postmenopausal phase, marked by a decline in estrogen levels, augments oxidative stress and free fatty acid levels, thereby heightening women’s vulnerability to metabolic syndrome and insulin resistance [68–70]. This hormonal transition culminates in an escalated risk for CVD [69, 70]. Similarly, testosterone was found to play even a minor cardioprotective role when it came to aging men and an extent aging women. As men age, hypogonadism correlates with lowered testosterone levels due to advanced age [71]. Although women have lesser amounts of testosterone throughout their lives, it also decreases as they age. It was discovered in a study [72] that lower levels of testosterone were associated with CAD in postmenopausal women. Low testosterone was shown to be attributed to a high incidence of CAD in men. A study revealed that at 40 years old, men who had serum testosterone levels under the suggested level also had a higher mortality risk due to CVD [71]. Similarly, in the case of COVID-19, estrogens enhance both innate and adaptive immune responses, potentially leading to faster pathogen clearance, milder symptoms, and stronger vaccine responses in women. Additionally, estrogen decreases the expression of angiotensin-converting enzyme 2 (ACE2) receptors, which are crucial for SARS-CoV-2 entry into host cells. Conversely, testosterone suppresses immune function, possibly explaining men’s greater susceptibility to infectious diseases. Furthermore, declining testosterone levels in aging men are linked to increased proinflammatory cytokines, raising the risk of severe COVID-19 progression and outcomes in older men [61].

Gender-based structural differences in the heart further contribute to the complexity of cardiac aging. Women typically possess smaller hearts with thicker walls and smaller chambers [73, 74]. This structural distinction may influence the onset of conditions like heart failure, as smaller hearts may lack adequate reserve capacity to withstand age-related changes [74]. Despite these challenges, data suggest a more favorable long-term prognosis for heart failure in women compared to men [75–77]. Both men and women experience worse health outcomes with extremes of body mass index and older age. Like, obesity has a greater impact on COVID-19 outcomes in women, while the influence of older age on outcomes is more significant in men [78]. Similarly, aging process also manifests differently in the blood vessels of men and women. Premenopausal women generally exhibit a more gradual progression of atherosclerosis compared to men of analogous age [79, 80]. Postmenopausal women, conversely, tend to have stiffer arteries compared to their male counterparts [81]. This vascular aging pattern is partially attributed to the protective influence of estrogen on vascular health. In light of estrogen’s impact on the heart and vasculature, HRT emerges as a potential strategy to offset the augmented CVD risk accompanying declining estrogen levels in postmenopausal women [81]. However, the HRT debate continues, emphasizing the necessity for individualized decision-making [59].

Gender significantly influences COVID-19 and cardiac aging, impacting both the risk and progression of cardiovascular diseases. COVID-19 infection is linked to heightened short- and long-term risks of CVD and mortality. Continual monitoring of signs and symptoms indicating the development of these cardiovascular complications post-diagnosis and for at least one year post-recovery could be advantageous for infected patients, particularly those who experienced severe illness [82]. In addition to COVID-19 infection, COVID-19 mRNA vaccines have also demonstrated effects on cardiac health. Myocardial injury, presenting clinically as myocarditis, has recently emerged as a potential severe adverse event following COVID-19 mRNA vaccine administration, predominantly affecting young men within a few days post-vaccination [83]. The intricate interplay of hormones, particularly estrogen, alongside structural differences, crafts a landscape necessitating gender-specific approaches to prevention and treatment. Despite the current limitations in research advancement, the ongoing exploration of COVID-19 infection and its effect on gender and cardiac aging is paramount. As we continue to demystify the complexities of COVID-19, gender and cardiac aging, the development of tailored strategies addressing each gender’s unique needs is necessary, ultimately enhancing cardiovascular health outcomes for all individuals.

## Influence of ethnic orientation on cardiac agedness

COVID-19 has had devastating effects on global healthcare systems, especially impairing the elderly and individuals with chronic comorbidities who are at particularly high risk of mortality and morbidity [84]. Studying the association between COVID-19 severity and noncommunicable diseases such as hypertension, diabetes, and cardiovascular disease in ethnic groups is crucial. Cardiac aging is intricately intertwined with diverse elements including genetic predisposition, lifestyle choices, and healthcare accessibility. These factors are deeply rooted in ethnic orientation and play a crucial role in shaping the trajectory and impact of cardiac aging across various populations. The growing evidence highlighting the escalating prevalence of CVD and accelerated cardiac aging within minority groups underscores the critical need for a nuanced, ethnically sensitive approach to cardiac health management [85].

The COVID-19 pandemic has underscored disparities among at-risk populations. Black and Hispanic patients have borne a disproportionate burden in terms of hospitalizations, morbidity, and mortality compared to non-Hispanic Whites [86]. Although death rates from COVID-19 were highest among Hispanic populations, the most significant mortality increases occurred in the Black population, largely attributed to heart disease and diabetes rather than COVID-19 itself [87]. Previous studies have demonstrated a higher disease burden, lower rates of bystander cardiopulmonary resuscitation (BCPR), and poorer outcomes among Black and Hispanic patients following out-of-hospital cardiac arrest (OHCA). Female OHCA patients also exhibit lower BCPR rates compared to males, with differences in survival outcomes. However, the impact of the COVID-19 pandemic on OHCA incidence and outcomes among different health disparity populations remains uncertain [88]. Another previous study found that non-Hispanic Black, Hispanic, and non-Hispanic Asian populations experienced a significantly higher rise in deaths related to heart and cerebrovascular diseases during the COVID-19 pandemic compared to the non-Hispanic White population [89]. Before the onset of the COVID-19 pandemic, structural and social factors, including housing and job insecurity, as well as high poverty rates, disproportionately affected racial and ethnic minority populations. These factors contributed to the higher burden of CVD observed in these communities. The effects of the COVID-19 pandemic are likely to have worsened these social disadvantages, leading to increased CVD mortality among specific racial and ethnic populations [90]. Not only general population but healthcare workers, especially those from ethnic minority backgrounds, have also been disproportionately higher risk of Severe Acute Respiratory Syndrome Coronavirus 2 infection compared to the general population [91]. Due to the unequal impact of COVID-19 on minority communities, it’s imperative to include ethnic minority populations in COVID-19 trials. This inclusion is essential for comprehending variations in intervention effects on disease severity and outcomes, and for addressing significant knowledge gaps [92]. Also, achieving vaccine equity requires multifaceted policies and programming that respect community concerns, prioritize informed deliberation, invest in community-based engagement, improve accessibility and transparency of information, and reduce structural barriers to vaccination [93].

The role of exercise in mitigating cardiac aging and preventing COVID-19 infection emerges as a significant area of focus. The research underscores the transformative impact of exercise training in reversing cardiac aging signs, even in populations with a high predisposition to heart failure with preserved ejection fraction, a prevalent subtype of heart failure in aging adults [94]. Similarly, literature indicates that engaging in physical activity plays a significant role in preventing and treating COVID-19. It can aid in the recovery of physical function, alleviate symptoms of post-acute COVID-19 syndrome, and enhance patients’ psychological well-being [95]. Despite the proven efficacy of exercise training and cardiac rehabilitation in enhancing cardiac quality of life and reducing hospitalization rates [96], ethnic disparities in physical activity levels persist, as noted by the CDC [97]. Healthcare access is known to be a linchpin in the modulation of cardiac aging that further accentuates these ethnic disparities. A diminished likelihood of cardiac rehabilitation referrals among minority populations, including Black, Hispanic, and Asian individuals, compared to their white counterparts, highlights the systemic barriers faced by these communities [98]. This healthcare access gap, coupled with the pivotal role of early detection and timely interventions in cardiac health maintenance, underscores the imperative to dismantle these barriers to ensure equitable cardiac health outcomes across diverse populations. The intricate mosaic of cardiac aging, painted with strokes of genetics, lifestyle, and healthcare access, is further complicated by the overlay of ethnic disparities. The heightened vulnerability of certain ethnic groups to accelerated cardiac aging and associated cardiovascular conditions necessitates a comprehensive, culturally sensitive approach to cardiac health management. This approach should encompass targeted interventions addressing unique ethnic risk factors, promotion of physical activity, and enhancement of healthcare accessibility and equity. By ensuring a holistic, inclusive, and effective strategy for diverse populations, we can pave the path towards optimal cardiac health and well-being for all individuals, irrespective of their ethnic orientation.

In the United States, differences in cardiovascular risk are mainly linked to socioeconomic status (SES) rather than race or ethnicity. It’s important to focus on lifestyle counseling and early screening for risk factors among socioeconomically disadvantaged individuals, regardless of their racial or ethnic background, to help reduce disparities in cardiovascular health outcomes [99]. Since CVD is the leading cause of death in the United States, with significant disparities in CVD-related illness and mortality. Marginalized racial and ethnic groups, such as Black Americans, Indigenous People, South and Southeast Asians, Native Hawaiians, and Pacific Islanders, are generally at higher risk for CVD and the development of traditional risk factors, compared to non-Hispanic Whites. Additionally, many of these groups face adverse social determinants of health, particularly lower SES, which may contribute to accelerated arterial aging, although South Asians typically experience higher SES [100].

Communities of color face significant disparities in access to and quality of CVD care. A major barrier is the lack of health insurance, with nearly 7.3 million nonelderly adults with CVD in the U.S. uninsured in 2018 [101]. Despite improvements from the Affordable Care Act, uninsured rates remain 2 to 4 times higher among Hispanic (28.7%) and Black (12.9%) individuals compared to White individuals (7.4%) [102]. Racial health inequities are reinforced by discriminatory policies at both state and federal levels that are ingrained in the healthcare system. Evidence shows that hospitals serving underserved communities face higher financial penalties for lower quality care scores, such as readmission rates. These metrics often overlook critical social risk factors that significantly influence health outcomes in these populations [103].

Cultural differences in diet and lifestyle, along with genetic predispositions, play a significant role in cardiac aging among ethnic groups. For instance, traditional diets rich in fruits, vegetables, and whole grains, as seen in Mediterranean and Asian cuisines, may promote better heart health, while diets high in processed foods can increase cardiovascular risk. Additionally, genetic factors can influence metabolic processes and susceptibility to heart diseases, leading to variations in cardiac aging. These combined influences underscore the importance of culturally tailored health interventions to address disparities in cardiovascular health outcomes.

## Metabolic disorders and cardiac dysfunction

The interplay between COVID-19 infection, diabetes, and cardiac aging involves a complex array of pathophysiological mechanisms, leading to significant morbidity and mortality. While the risk of COVID-19 infection is elevated in individuals diagnosed with diabetes compared to those without, there is a lack of research on the risk of cardiovascular disease CVD in COVID-19-infected patients with diabetes compared to those without COVID-19 infection [104]. Emerging evidence indicates that COVID-19 infection may lead to incident CVD in the long term, with chronic conditions like diabetes potentially aggravating this risk. A recent study explored the risk of CVD after COVID-19 diagnosis in adults with and without diabetes, revealing a significantly higher post-acute risk of incident cardiovascular outcomes among patients with COVID-19, irrespective of diabetes status. Thus, ongoing monitoring for new-onset CVD may be crucial beyond the initial 30 days following a COVID-19 diagnosis [105].

Another study revealed that pregnant women with comorbidities such as diabetes mellitus, hypertension, and cardiovascular disease faced heightened risks for severe COVID-19-related outcomes, maternal complications, and adverse birth outcomes. Additionally, the study identified lesser-known risk factors, including HIV infection, prepregnancy underweight, and anemia. Despite pregnant women being recognized as a high-risk population, those with these additional risk factors warrant special attention for prevention and treatment [106]. Patients with underlying metabolic dysfunction, such as type 2 diabetes mellitus and obesity, face an elevated risk of COVID-19 complications, including multi-organ dysfunction, due to a disrupted immune response and cellular energy deficiency. These individuals experience chronic inflammation, heightening susceptibility to severe immune reactions triggered by the hypoxic cellular environment and cytokine storm associated with COVID-19. The altered metabolic profile and energy production of immune cells further contribute to an imbalanced immune response. Understanding these critical immune-metabolic interactions may aid in developing effective treatments for COVID-19 [107].

Roy and colleagues examined recent literature on the association between CVD and diabetes mellitus in COVID-19 infections, highlighting potential mechanisms. Despite lacking prior CVD history, COVID-19 patients can develop complications such as myocardial injury, cardiomyopathy, and venous thromboembolism following infection with severe acute respiratory syndrome coronavirus 2 (SARS-CoV-2), requiring emergency clinical support for management. While the link between diabetes and severe COVID-19 complications remains unclear, limited data suggest that markers such as interleukin (IL)-1, IL-6, C-reactive protein, and D-dimer are associated with COVID-19 severity in diabetic individuals [108]. In another study, by Bioinformatic analysis the important Genes and their key functions in Type 2 diabetic hearts were identified. Patients with type 2 diabetes (T2D) and SARS-CoV-2 infection develop acute cardiovascular syndrome, necessitating understanding of underlying mechanisms. Bioinformatic analysis of public datasets identifies pathogenic and prognostic genes in T2D hearts, revealing CAPNS1 as a crucial gene. CAPNS1 upregulation in T2D hearts is associated with calpain/CAPNS1-mediated Junctophilin2 (Jp2) hydrolysis and nuclear translocation, suggesting CAPNS1 as a potential therapeutic target for adverse prognostics in T2D patients with SARS-CoV-2 infection [109].

In addition to standard post-COVID-19 assessments, patients with an elevated metabolic risk profile should undergo supplementary evaluation by a cardiologist, which may include comprehensive echocardiography. This evaluation should be conducted both during the acute infection phase and throughout the recovery period [110]. Vitamin D is crucial for immune function and has anti-inflammatory properties, which could be significant in the context of CVD and COVID-19. However, current studies have shown limited benefits of vitamin D supplementation in COVID-19 patients, with no specific research on those with CVD and related complications. Additionally, while vitamin D offers protective effects on the cardiovascular system, such as enhancing myocardial contractility and anti-thrombotic effects, it remains uncertain whether vitamin D supplementation can alleviate CVD complications associated with COVID-19 [111]. Malnutrition is recognized to heighten susceptibility to viral infections and disease progression. Thus, considering the nutritional status of diabetic patients and providing appropriate supplementation of essential nutrients can help alleviate the symptoms of COVID-19 in individuals with diabetes mellitus [112].

## Inflammatory pathways and oxidative stress

The role of inflammation and oxidative stress in diabetic cardiomyopathy is a topic that is being highly researched [113]. Systemic inflammation has been recognized as both a cause and an outcome of diabetes [114–116]. Recent findings indicate that the activation of Cannabinoid 2 receptor (CB2R) signaling pathways in mitigating myocardial dysfunction suggests that pharmacological activation of CB2R can attenuate diabetes-induced inflammation, oxidative/nitrative stress, fibrosis, and cell death, resulting in the preservation of cardiac function [113]. This indicates a potential therapeutic target for managing cardiac complications in diabetes.

## SGLT2 and cardiac inflammation

Mroueh et al. (2023) advance this understanding by evaluating the expression of Sodium-Glucose Co-Transporter 2 (SGLT2) in the left ventricle of cardiac patients. Their study establishes a correlation between SGLT2 mRNA expression and pro-inflammatory markers, suggesting a role for SGLT2 in heart failure associated with diabetes. This finding is particularly relevant given the emerging role of SGLT2 inhibitors in the management of heart failure [117]. These recent studies highlight the potential of CB2R activation and SGLT2 inhibition in mitigating the cardiac complications of diabetes, offering hope for improved clinical outcomes in this vulnerable population.

## Overview of long-term effects of COVID-19 on cardiac health and cardiac aging

**Figure 4:**
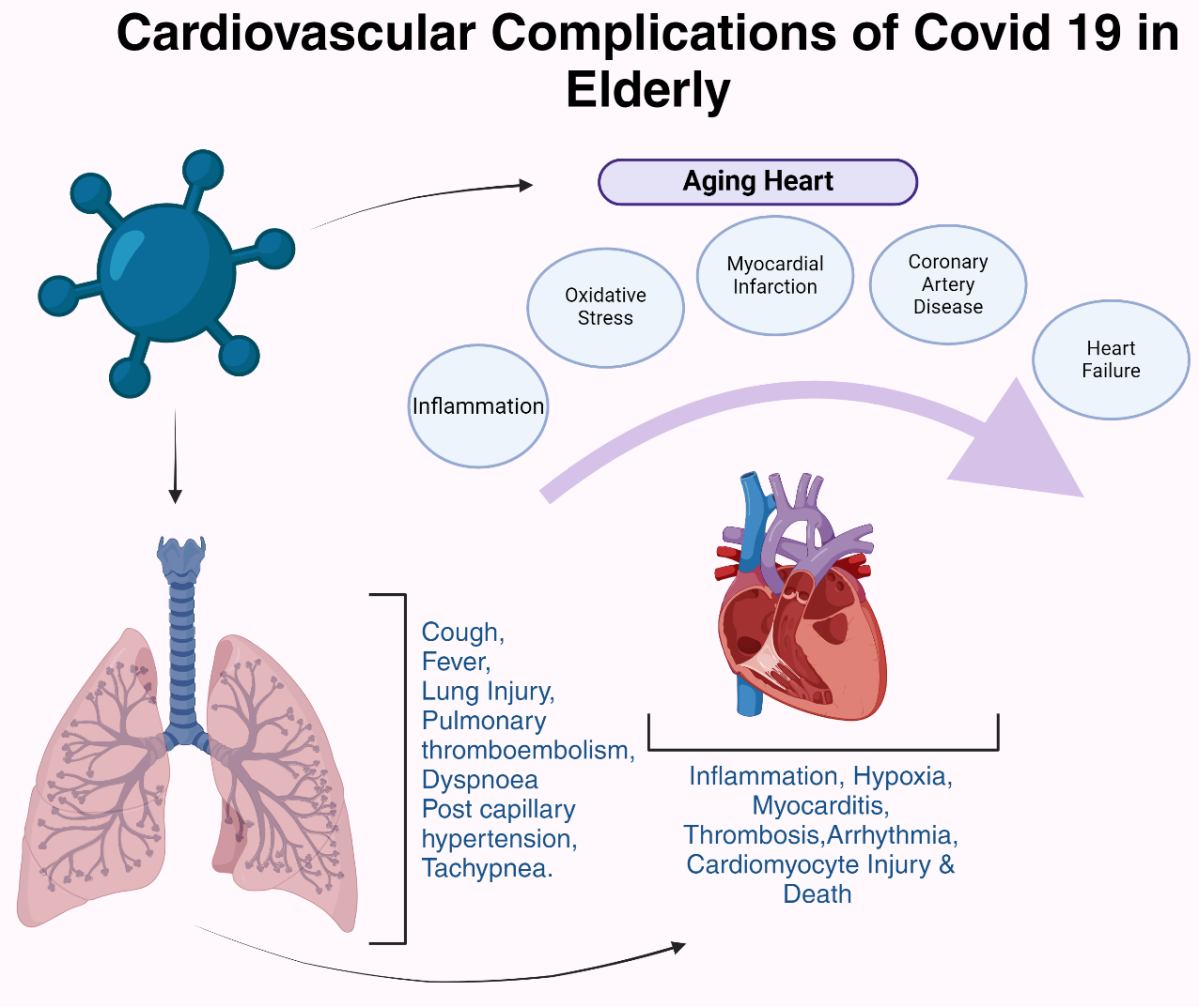
**Cardiovascular complications of COVID-19 in elderly people.** COVID-19 exacerbates cardiac injuries, especially in older patients with a cardiovascular medical background, increasing the likelihood of developing critical conditions.

The COVID-19 pandemic, caused by the SARS-CoV-2 virus, has manifested a colossal number of clinical complications that extend beyond the respiratory system. There is emerging evidence that highlights significant cardiac implications (Figure 4). The long-term effects of COVID-19 on cardiac health and cardiac aging are areas of flourishing research, due to the many clinicians and scientists who endeavor to understand and mitigate these impacts.

## Adverse cardiac effects of SARS-CoV-2 infection

The SARS-CoV-2 virus has been identified as a significant threat to cardiovascular health due to the ability of the virus to directly infiltrate myocardial cells, setting off a cascade of detrimental cardiac events including myocarditis, myocardial infarction, and heart failure [118]. Additionally, the systemic inflammatory response elicited by the virus can exacerbate pre-existing cardiac conditions and contribute to the onset of new cardiovascular complications (Figure 4). The body’s effort to combat viral infection leads to a widespread inflammatory response, which can inadvertently exacerbate pre-existing cardiac conditions. Individuals with a history of cardiovascular diseases may find their conditions worsening under the strain of the body’s immune response to the virus and the inflammation can lay the groundwork for new cardiovascular complications, even in previously healthy individuals, by contributing to the buildup of plaque in the arteries, disrupting blood flow, and leading to arrhythmias [118]. The long-term consequences of these effects remain a significant concern, necessitating ongoing monitoring and management of COVID-19 survivors for potential cardiac sequelae [118]. The inflammatory damage inflicted on the heart and the vascular system may persist, leading to chronic cardiac conditions, endothelial dysfunction, microvascular damage, reduced cardiac function, and an increased risk for future cardiac events [119].

The SARS-CoV-2 binding site has increased affinity for the angiotensin I converting enzyme 2 (ACE2) receptor which is expressed in the lung and vascular endothelial cells [120]. It has yet to be established whether a higher rate of cardiac injury with increased age is due to viral injury or a crushing immune response within myocardium, or both. It is typical of aging systems to have a reduction in circulating levels of CD4+ and CD8+ T lymphocytes and a decreased capability to phagocytose apoptotic cells by aging macrophages may induce a vascular pro-inflammatory state [120]. This imbalance is exacerbated with SARS-CoV-2 infection leading to further deficiency of CD4+T cells and macrophage response. Therefore, elderly patients tend to have reduced viral clearance, which in turn generates a cytokine storm [120].

## Therapeutic strategies to improve cardiac health

The human heart’s remarkable endurance and resilience are continuously challenged by a myriad of physiological and external stressors throughout life. Despite its robust nature, the heart’s capacity for regeneration and repair is notably limited, particularly in the adult stages of life. This limitation underscores the critical need for innovative therapeutic strategies aimed at enhancing cardiac regeneration and mitigating damage inflicted upon the myocardial architecture. Research findings in CVD significantly influence treatment strategies and preventive measures by providing evidence-based insights into the pathophysiology, risk factors, and effective interventions for these conditions. For instance, identifying genetic predispositions and lifestyle factors such as diet, exercise, and smoking can lead to personalized medicine approaches and targeted interventions. Additionally, advancements in understanding the molecular mechanisms of CVD can result in the development of novel drugs and therapies, improving patient outcomes. The gap between research and clinical practice can be bridged by adopting the following strategies: clinical research should focus on practice-oriented questions, involving practitioners in setting the agenda and formulating research questions; the divide between empiricist and constructivist positions, as well as between quantitative and qualitative methods, can be resolved through epistemological and methodological pluralism; clinical research should be guided by a developmental perspective; and collaboration between practitioners and researchers should occur at both programmatic and institutional levels [121].

## Macrophages: key to enhanced cardiac regeneration

The adult heart is incapable of regenerating itself after an injury and instead, the extensive cardiomyocyte death is followed by scar formation that can reconstruct the myocardial architecture. However, neonatal hearts are capable of regenerating after an injury. Since the 1960s, newborn patients who survived an MI soon after birth were able to restore much structural and functional capabilities within weeks. Regenerative hearts were found to have retained macrophages for a longer period following an injury [122]. The cardiomyocytes of neonates contained a greater proportion of proliferating cardiomyocytes in comparison to those of older adult patients, suggesting that cardiac regeneration, while possible in neonates, is an ability that is lost with age [123]. In the case of myocardial infarction, macrophages play a role in mitigating the injury and scavenging cellular debris through the secretion of cytoprotective factors that reduce the inflammatory response, particularly by suppressing myofibroblast activation and fibrosis [124]. While the reason why macrophages are a crucial part of the heart’s regenerative response is not fully understood, several pathways, including axonal regrowth, angiogenesis, ECM degradation, and efferocytosis, commonly involve macrophages and are activated during the process of regeneration [124]. In one study, examining the shifts in macrophage population concerning age and disease in mice, it was found that depletion of CX3R1+ macrophages before an MI led to impaired healing, reduced cardiac function, and increased mortality [125]. Considering the importance of macrophages’ role in regeneration, therapies related to modifying adult macrophage populations can potentially be used to enhance repair.

## Exosomes in cardiac care

Exosomes, which are a type of extracellular vesicle capable of being produced by each cell type under stressful conditions like ischemia, also have the potential to be used as a therapeutic agent [126]. Exosomes delivered *in vivo* have been found to restore cardioprotective effects, including the suppression of proinflammatory cytokine expression and efferocytosis [126]. Additionally, an RNA component of the cardiac exosome, EV-YF1, increased IL-10 expression and protection against ischemia when delivered to macrophages [127]. Exosomal miRNAs have also been shown to be crucial to exosome therapy. miRNA-146a, which is down-expressed in cardiovascular aging, has had protective effects after an MI injury, along with miR-181b, which can protect cardiac function and reduce infarction size, but is often decreased in the aging aorta [128]. However, delivering an adequate amount of exosomes to macrophages is often found to be challenging as they can instead be taken up by macrophages or other immune cells other than the macrophages at the site of injury [129]. As a result, strategies have been developed to create a more streamlined way of delivering exosomes. In one study, cloaking, a modification that is directly applied to the exosome rather than through a producer cell, resulted in the localization of exosomes to the site of ischemic myocardium [130]. Exosomes were cloaked with a biotin-tagged ischemic peptide and were found in higher quantities within the infarcted region of the heart compared to exosomes without the cloak [130].

## Targeting reactive oxygen species

Reactive oxygen species (ROS) are directly involved with the dysfunction of cardiac tissues by promoting myocardial growth, extracellular matrix remodeling, and activating hypertrophy and apoptotic signaling pathways [131]. An excess of ROS in ryanodine receptors within the sarcoplasmic reticulum increases the activity of (RyRs), thus increasing the release of calcium from the SR during excitation-contraction coupling, and decreasing the activity of SR calcium-adenosine triphosphatase 2 (SERCA2), resulting in a calcium overload [132]. Mitochondria also serves as a source of ROS, particularly from the electron transport chain complexes. ROS can impair ATP levels, which was seen when looking at the status of energy metabolism in the context of a myocardial infarction [133]. Using ROS as a target has been shown to suppress the cardiac aging process, with aged cardiac stem cells being transformed into a younger phenotype upon the reduction of ROS generation [134]. Another pathway for reducing ROS production is through the inhibition of xanthine oxidase. The activity of SERCA2a and Ca transient channels is boosted following the use of a xanthine oxidase inhibitor as well, hence preventing calcium overload [135]. Lasers have also been used to correct mitochondrial function, resulting in reduced oxidative stress, and leading to improved systolic dysfunction and reduced infarct size [136]. The consumption of foods rich in antioxidants has also been found to reduce levels of oxidative stress markers like MDA and the incidence of cardiac remodeling, however, evidence of the benefit of antioxidant therapies in clinical trials is limited [131].

## Stem cells and senescence

Mesenchymal stem cells (MSCs) are heterogeneous stromal cells originating from the mesoderm and ectoderm [28]. They contain multipotency and regenerative characteristics and evidence supports their role in maintaining tissue homeostasis, and preventing aging-related diseases, and have been used in clinically relevant therapies [137]. The human heart relies on its stem cells to replace aging or dying cells [138] In human cardiac stem cells, dysfunctional telomeres are biomarkers for aging and heart failure. The shortest telomere, rather than the mean telomere length, determines the function and fate of the cell [139]. As individuals age, the capacity of their stem cells to replicate, regenerate, and proliferate diminishes. Concurrently, the stem cells show increased cell cycle arrest, oxidative stress injury, and a decline in immunomodulatory capacity and paracrine effects [137]. Cellular senescence, characterized by irreversible cell cycle arrest, can be triggered by various factors, including DNA damage, telomere shortening, and stress. While senescence serves as a tumor-suppressive mechanism by preventing the proliferation of damaged cells, it also contributes to aging and age-related diseases [140]. Evidence shows that senescent cells, although in cellular arrest, remain metabolically active. The senescence-associated secretory phenotype (SASP), secretes factors, including proteases, that detrimentally affect the tissue microenvironment. Proteases secreted by SASP can target the extracellular matrix, leading to alternations in tissue structure. This, in turn, can potentially diminish tissue tension and elasticity [140].

The first approach to slowing, preventing, and possibly even reversing the senescence of MSCs is targeting their genetics [141]. Epigenetic reprogramming involves the modification of both coding and noncoding RNA epigenetics, which is a promising direction. By targeting RNAs either directly or indirectly involved in MSC senescence, their effects might be mitigated [141]. Additionally, miRNAs are small molecules that interfere with transcription in many ways, a primary way being interaction with the target gene RNA [142]. It has been shown that miR-195, significantly increased TERT expression and that miR-195 inhibition significantly induced telomere lengthening in aged MSCs [143]. Building upon this discovery, the inhibition of many additional miRNA molecules (miR-141-3p, mi-34a, miR-155, etc.) was found to slow the senescence of MSCs [144–146]. Finally, senescence-associated secretory phenotype (SASP) factors are involved in propagating inflammation responses and contribute to the aging and senescence of nearby cells [143].

## Lifestyle changes to improve cardiac health span

**Figure 5:**
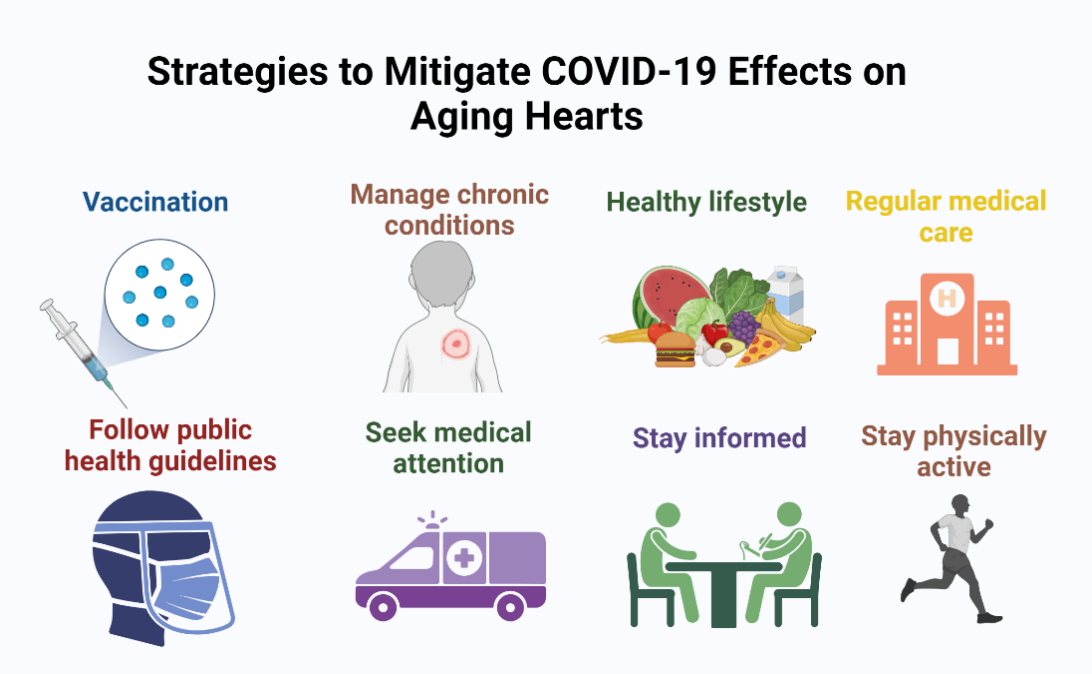
To prevent the impact of COVID-19 on aging hearts, implement measures such as vaccination, adherence to public health guidelines, managing chronic conditions, staying active, and seeking regular medical care.

Separate from small molecule therapies to combat cardiac aging are interventions aimed at improving metabolic function. These therapies include dietary modifications, aerobic and resistance training, as well as some supplementation therapies [147]. Caloric restriction, while avoiding malnutrition, has been associated with increased life span and improved metabolic health in multiple model systems [148]. The CALERIE study demonstrated that calorie restriction in humans was associated with weight loss, a reduction in total daily energy expenditure (TDEE), and a reduction in inflammatory markers [149] (Figure 5). Regular exercise has also been shown to reduce central body fat, improve muscle mass and function, and increase insulin sensitivity compared to sedentary adults [150, 151]. Exercise has also been shown to increase the activation of satellite cells and improve progenitor cell function [152]. Resistance training has also been shown to increase skeletal muscle mass and function, however, older adults will need to increase protein intake compared to younger individuals to achieve similar results [153]. Finally, in addition to behavioral modification, some pharmacologic supplementation has also been shown to be effective. Leucine supplementation and b-hydroxy-b-methylbutyrate (HMB) has been shown to increase muscle mass and function in conjunction with resistance training [154]. Omega-3 fatty acids have also shown improved muscle mass and function with aging; however, their mechanism of action is not well understood [155].

## Long term effects of COVID-19 on cardiac health

**Figure 6:**
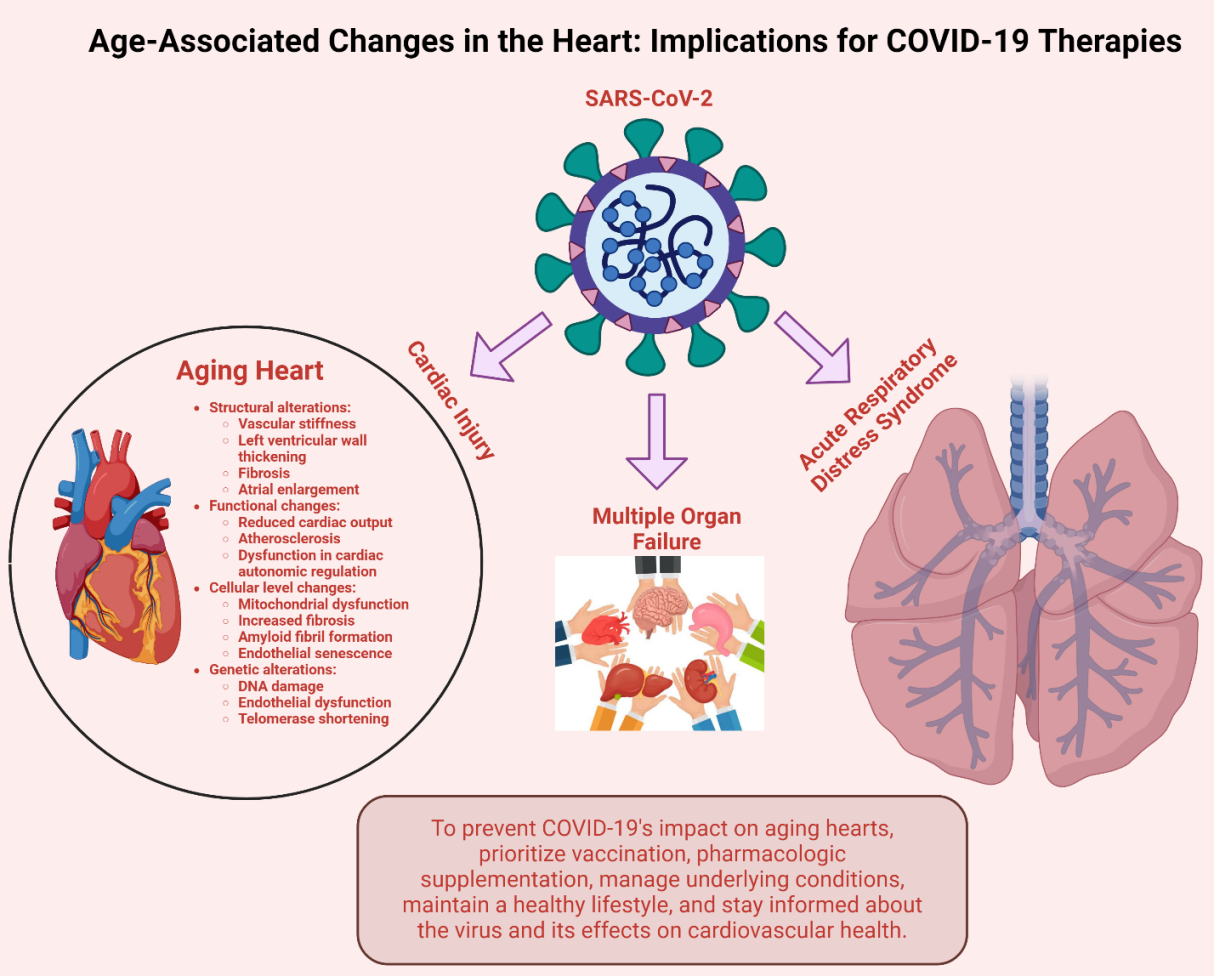
**Ways COVID-19 contributes to cardiac aging.** A COVID-19 infection can cause macroscopic and microscopic altercations that induce cardiovascular dysfunction. Image created by Biorender.com.

The COVID-19 pandemic resulted in cardiac complications, adverse lifestyle alterations during lockdowns, and constraints in preventive, diagnostic, and therapeutic measures. The cardiac issues arising from acute COVID-19 have been extensively documented, yet the subsequent cardiovascular effects of COVID-19 in the post-acute phase remain inadequately characterized. The cardiovascular effects of COVID-19 exhibit pathophysiological, consequential, and time-dependent patterns, encompassing myocardial injury, thrombotic events, heart failure, and arrhythmias. These range from acute COVID-19 cardiovascular syndrome to short-term and long-term post-COVID-19 manifestations [156–158]. Using US Department of Veterans Affairs national healthcare databases, Xie and colleagues constructed a cohort of 153,760 COVID-19 individuals and two control cohorts totaling 11,496,058 individuals. The study found that beyond 30 days post-infection, COVID-19 patients faced increased risks of various cardiovascular outcomes, irrespective of hospitalization status. These risks escalated based on the level of care during the acute phase, emphasizing the significant long-term burden of cardiovascular disease post-COVID-19, warranting attention in survivor care pathways [156]. Similarly, Loboda and team conducted a study in which at approximately 4 months post-infection, they evaluated cardiac complications, exercise capacity, blood pressure, echocardiography, Holter monitoring, and lab results (cholesterol, glucose, creatinine) in convalescent individuals. Using the Systemic Coronary Risk Estimation 2 algorithm, they estimated the 10-year risk of fatal and nonfatal atherosclerotic cardiovascular events, revealing a small number of post-COVID-19 cardiac issues, particularly in men, alongside a notable risk of atherosclerosis-related diseases. Hence recommended that medical assessment for COVID-19 survivors should include managing atherosclerosis risk factors [159] (Figure 6).

Socioeconomic status (SE) significantly predicts early morbidity and mortality in overall health, with lower SE correlating with heightened CVD mortality and poorer CVD risk profiles. COVID-19 severity and mortality are elevated in individuals with CVD comorbidity, as both conditions share critical risk factors. The pandemic disproportionately impacts those of lower SE and ethnic minority backgrounds, with mortality rates doubling in the most deprived regions compared to the least deprived ones [160]. For instance, African Americans (AA) have faced a disproportionate impact from COVID-19, accounting for 30%-60% of deaths despite comprising only 13% of the US population. Preliminary evidence indicates that pregnant women and individuals with CVD may encounter more severe outcomes from severe coronavirus infection [161]. Similarly, gender disparities exist, contributing to underlying causes and predictors of immediate and short-term cardiovascular readmissions following COVID-19 hospitalizations in the USA. Vardar and team’s research revealed that among COVID-19 patients requiring hospitalization, factors such as male gender, as well as underlying renal, pulmonary, and liver conditions, correlate with increased cardiovascular readmission risk. These findings offer valuable insights for healthcare systems to develop strategies aimed at reducing readmissions [162]. With various previous studies we have learnt that cardiac injury was prevalent during acute COVID-19, linked to poorer short-term outcomes, and its etiology is multifaceted, with imaging abnormalities often present regardless of acute illness severity. Yet, there is still much to uncover about the progression and clinical significance of these findings in survivors, particularly those experiencing post-acute COVID-19 syndrome [163].

In addition to cardiovascular disease, diabetes and hypertension are significant risk factors for severity and mortality in individuals infected with COVID-19. These conditions should be a primary focus in managing this infection [164]. Current evidence indicates that COVID-19 infection can lead to hyperglycemia, ketoacidosis, and occasionally new-onset Type 1 diabetes, while also worsening prediabetes and existing Type 2 diabetes. Since the onset of the pandemic, it has become clear that individuals with metabolic diseases face a higher risk of severe COVID-19 and increased mortality compared to those without these conditions. Furthermore, COVID-19 infection may exacerbate metabolic disorders and is associated with a higher prevalence of long-COVID among diabetes patients, who tend to experience more long-term effects. The underlying mechanisms for these differences are not yet fully understood and warrant further research in the coming years [165]. As the COVID-19 pandemic significantly altered daily routines worldwide due to lockdowns, impacting various factors that worsen blood pressure (BP). While some changes, like increased sleep and reduced pollution, could lower BP, other behaviors—such as higher alcohol consumption, smoking, decreased physical activity, and poor medication adherence—likely contributed to increased BP levels [166]. A study by Akpek and colleagues examined COVID-19 impact on BP in 153 hospitalized patients, revealing that both systolic and diastolic BP were significantly higher post-infection compared to admission. New onset hypertension occurred in 18 patients within an average follow-up of about 32 days, suggesting that COVID-19 may lead to increased BP and new hypertension cases [167]. In addition to impacting the lungs, COVID-19 can damage the cardiovascular, digestive, urinary, hepatic, and central nervous systems. Beyond its immediate effects, the virus may lead to long-term complications as well [168].

## CONCLUSIONS

The journey of cardiac aging is intricate, shaped by genetic, environmental, and physiological factors, posing a significant challenge for scientific and medical communities. Understanding the cardiovascular system’s structure and function reveals its essential role in sustaining life through the delicate balance of oxygen, nutrients, and waste removal. However, oxidative stress, mitochondrial dysfunction, and impaired autophagy contribute to the gradual decline of cardiovascular health with age, highlighting the need for a comprehensive understanding of cardiac aging mechanisms. Gender and ethnicity further influence cardiac aging, with estrogen providing a protective shield for women’s cardiovascular health that diminishes post-menopause. Ethnic predispositions and cultural lifestyle elements add complexity, emphasizing the need for personalized cardiac care approaches. The COVID-19 pandemic amplifies cardiac health concerns, revealing severe cardiovascular complications in adults and children alike. Monitoring, managing, and mitigating these impacts require sustained research, clinical observation, and healthcare strategies. Innovative therapeutic strategies, including cardiac regeneration and myocardial damage alleviation, hold promise, with potential roles for macrophages, exosomes, and reactive oxygen species targeting. A comprehensive understanding of the diverse influences on cardiac aging is crucial for targeted interventions. Integrating insights into structural, functional, and external influences on cardiac aging is essential for promoting cardiac health in an aging global population, representing a societal commitment to enhancing longevity and quality of life. Moreover, a multidisciplinary approach is essential to effectively tackle cardiovascular diseases. By integrating insights from genetics, molecular biology, public health, and geriatrics, we can gain a more holistic understanding of CVD and develop innovative solutions. This comprehensive perspective allows for personalized treatment strategies, targeted prevention programs, and improved patient outcomes, addressing the complex interplay of factors that contribute to cardiac health. Collaboration across these diverse fields is crucial for advancing research and implementing effective interventions to reduce the global burden of cardiovascular diseases.

## Future Directions

Technological advances in recent years have enabled us to assist a broader range of patients with complex cardiac and coronary anatomy. With novel devices and advanced imaging techniques, we can now treat more intricate coronary conditions and address valvular diseases in patients with multiple comorbidities. These advancements in interventional cardiology will continue to shape our practice in the future, making their widespread availability to sicker patients crucial [169]. Future research directions for addressing cardiac health in aging populations post-COVID-19 include longitudinal studies to assess long-term cardiovascular effects, exploration of novel therapeutic interventions targeting cardiac regeneration and inflammation reduction, and investigation of gender-specific and ethnic disparities in cardiac outcomes. Implementing comprehensive cardiac rehabilitation programs tailored for aging individuals, exploring telemedicine and remote monitoring technologies, and examining socioeconomic determinants on cardiac health outcomes are crucial. Enhancing analysis of patients referred to cardiac rehabilitation by race, gender, socioeconomic status, and other factors at an institutional level can illuminate disparities, with potential for a standardized systematic approach to improve referral rates. Collaboration across disciplines is essential for developing evidence-based strategies to mitigate long-term cardiovascular risks and improve access to cardiac care for vulnerable aging populations impaired by COVID-19.

### Data availability

No data were used for the research described in the article.
